# The Brain of the Black (*Diceros bicornis*) and White (*Ceratotherium simum*) African Rhinoceroses: Morphology and Volumetrics from Magnetic Resonance Imaging

**DOI:** 10.3389/fnana.2017.00074

**Published:** 2017-08-31

**Authors:** Adhil Bhagwandin, Mark Haagensen, Paul R. Manger

**Affiliations:** ^1^School of Anatomical Sciences, Faculty of Health Sciences, University of the Witwatersrand Johannesburg, South Africa; ^2^Department of Radiology, Wits Donald Gordon Medical Centre, University of the Witwatersrand Johannesburg, South Africa

**Keywords:** rhinoceros, Rhinocerotidae, Perissodactyla, central nervous system, surface anatomy

## Abstract

The morphology and volumetrics of the understudied brains of two iconic large terrestrial African mammals: the black (*Diceros bicornis*) and white (*Ceratotherium simum*) rhinoceroses are described. The black rhinoceros is typically solitary whereas the white rhinoceros is social, and both are members of the Perissodactyl order. Here, we provide descriptions of the surface of the brain of each rhinoceros. For both species, we use magnetic resonance images (MRI) to develop a description of the internal anatomy of the rhinoceros brain and to calculate the volume of the amygdala, cerebellum, corpus callosum, hippocampus, and ventricular system as well as to determine the gyrencephalic index. The morphology of both black and white rhinoceros brains is very similar to each other, although certain minor differences, seemingly related to diet, were noted, and both brains evince the general anatomy of the mammalian brain. The rhinoceros brains display no obvious neuroanatomical specializations in comparison to other mammals previously studied. In addition, the volumetric analyses indicate that the size of the various regions of the rhinoceros brain measured, as well as the extent of gyrification, are what would be predicted for a mammal with their brain mass when compared allometrically to previously published data. We conclude that the brains of the black and white rhinoceros exhibit a typically mammalian organization at a superficial level, but histological studies may reveal specializations of interest in relation to rhinoceros behavior.

## Introduction

The fossil record of Perissodactyla places its earliest members in the upper Palaeocene (Radinsky, 1969), yet molecular studies suggest that the Perissodactyla diverged from Cetartiodactyla 97.5 – 83.4 million years ago (Eizirik et al., 2001; Price and Bininda-Emonds, 2009) or Carnivora approximately 80 million years ago (Springer et al., 2003). The order Perissodactyla is comprised of the Rhinoceritidae, Tapiridae, and Equidae families. The Rhinoceritidae includes five extant species: the black (*Diceros bicornis*) and white (*Ceratotherium simum*) African rhinoceroses, the Indian rhinoceros (*Rhinoceros unicornis*), the Javan rhinoceros (*Rhinoceros sondaicus*) and the Sumatran rhinoceros (*Dicerorhinus sumatrensis*) (Wilson and Reeder, 1993). After much debate regarding the phylogenetic relationships of these living species, it has been established that the Asian and African lineages diverged approximately 26 million years ago (Tougard, 2001).

The black and white rhinoceros are iconic species of the African continent with the white rhinoceros being the second largest terrestrial mammal after the African elephant. Observational studies of the African rhinoceroses have revealed some interesting behaviors, including aggression (Leuthold, 1977), a complex social structure (Owen-Smith, 1971), ear movements signaling intimidation (Leuthold, 1977), acute olfactory capabilities (Cave, 1966), and differences in the duration of a sleep bout between males and females (Santymire et al., 2012). For the most part, these behavioral studies do not refer to the structure (and inferred functional capacities) of the rhinoceros brain, as the information required to make this sort of interpolation is not available. Studies of rhinoceros brains would begin to unlock the neural architecture subserving the observed behaviors and may provide clues leading behavioral studies in new directions, providing a deeper understanding of rhinoceros behavior.

Very little is known about the structure, and through comparative implication, functional capacities, of the rhinoceros brain, although substantially more is known about the closely related horse brain (e.g., Yoshikawa, 1968; Cozzi et al., 2014). Most recorded observations detail the anatomy of the skull and only mention the anatomy of the brain in passing (Sparrman, 1778; Burchell, 1817). To date, drawings of the superficial appearance of the Indian and Sumatran/Javan rhinoceros brain (Owen, 1850; Garrod, 1878; Beddard and Treves, 1887), a few images of a fetal rhinoceros brain (Davies, 1952), the occasional report of brain mass or other measures in these species (Manger et al., 2010; Manger, 2011), and the topography of the retinal ganglion cells and visual acuity (Pettigrew and Manger, 2008; Coimbra and Manger, 2017) is all that is presently known of the rhinoceros brain. In the current paper we begin to fill this gap in our knowledge by providing descriptions of the external and internal structure of the brain of both the black and white African rhinoceros using direct observation of the brain surface and magnetic resonance imaging. We also provide volumetric analyses of various regions of the brain for both species and compare them to data previously published for other mammals.

## Materials and Methods

### Specimens

One brain of a black (*D. bicornis*) and a white (*C. simum*) African rhinoceros was used in the current study. A 40 year-old male black rhinoceros, obtained from the National Zoological Gardens, Pretoria, South Africa, was euthanized due to health complications following an intractable gastrointestinal illness, while the 3 year-old female white rhinoceros, obtained from Wildlife Assignments International, Hammanskraal, South Africa, was euthanized following irreparable damage to the right radial nerve after an attempted poaching incident. The animals were initially immobilized with a dose of etorphine (approximately 5 mg, i.m.), following which they were given an intravenous overdose of sodium pentobarbital (approximately 40 mg/kg, i.v.). Following cessation of the heartbeat, the head and neck was dissected free from the body and perfused using a gravity feed via the paired carotid arteries initially with a 20 l rinse of 0.9% saline to flush the remaining blood followed by 40 l of fixative (4% paraformaldehyde in 0.1M phosphate buffer) (Manger et al., 2009). The brains were then removed from the skull and post-fixed for 48 h in the fixative solution at 4°C, after which they were placed in a 30% sucrose in 0.1M phosphate buffer solution until equilibrated (approximately 7 days). Following this, the brains were placed in an antifreeze solution for 7 days at 4°C, before being stored at -20°C until use (Manger et al., 2009). Both animals were treated according to the guidelines of the University of the Witwatersrand Animal Ethics Committee (clearance certificate number 2008/36/1), which parallel those of the NIH for the use of animals in scientific experiments.

### Magnetic Resonance Imaging

The brains of both rhinoceroses were scanned in coronal, sagittal, and horizontal planes. The specimens were scanned on a Phillips 1.5 Tesla Intera System (Eindhoven, The Netherlands), using all three elements of the head and neck coil. The brains were removed from their containers, drained of excess fluid and placed in the head coil wrapped in a dry sheet, thus being exposed directly to air, which also partly entered the ventricles. After testing different scan parameters the following sequence was selected as giving the best detail and the least artifact (especially at the air-fluid interfaces). The selected T1 weighted inversion recovery sequence consisting of 2 mm slices without gap, had a TR (time to repeat) of between 6.5 and 10.9 ms and a TI (time to invert) of 300 ms. The number of signal averages varied between 3 and 4 with a flip angle of 90° and an echo train of 10. The scan times varied between 15 and 25 min. The antifreeze liquid in which the brains were stored showed high signal on both T1 and T2 weighted sequences and the routine clinical T1 and T2 sequences produced very similar T2 like images of the brain specimens. This is possibly related to the lack of water in the tissues of the specimen secondary to the fixation and storage process (see above). The images were processed using the freely available open source software program Osirix (Rosset et al., 2004)^1^.

### Volumetrics

In the current paper, the MRI scans allowed us to determine volume measurements for a variety of identifiable brain structures. These included the mid-sagittal cross-sectional area of the corpus callosum, the volume of the hippocampus, amygdala, cerebellum, and lateral ventricles and the gyrencephalic index (GI) for both species of African rhinoceros. We used the methodology of Manger et al. (2010) to calculate the volume of the corpus callosum, Patzke et al. (2015) to calculate the volume of the hippocampus and amygdala, Maseko et al. (2012) to calculate cerebellar volume, Maseko et al. (2011) to calculate the volume of the ventricular system and of Zilles et al. (1989); Pillay and Manger (2007), and Manger et al. (2012) to calculate the GI of the cerebral cortex.

## Results

The male black rhinoceros had a brain mass of 531 g while that of the female white rhinoceros was 536.5 g. Unfortunately, due to a lack of necessary heavy-duty equipment we were unable to obtain accurate measures of the body mass of each of these individuals. Previous reports indicate that the average body mass of a male black rhinoceros is 852 kg (Hitchins, 1968) and the average body mass of a female white rhinoceros is 1600 kg (Kirby, 1920). Using these average body masses, we could calculate the encephalization quotients (the relative mass of the brain compared to the mass of the body, using the regression provided in Manger, 2006) for each individual. The least squares regression provided by Manger (2006) was calculated using data from 271 mammal species, covering most mammalian orders, but excluded data from both primates and cetaceans, and did not use any phylogenetic corrections methods. Thus, the encephalization quotient for the male black rhinoceros was 0.469, while that for the female white rhinoceros was 0.299, both substantially below the expected encephalization quotient of 1 for mammals. The particularly low encephalization quotient for the female white rhinoceros may be due to the young age (3 years old) of this specimen. As we could not obtain a body mass from this specific specimen, we used the average adult female body mass to calculate the encephalization quotient as data on the average body mass of wild white rhinoceroses at different ages is not available. Thus, the encephalization quotients provided here must be interpreted cautiously when used in a comparative sense.

### Observable Features on the Surface of the Brain

**FIGURE 1:**
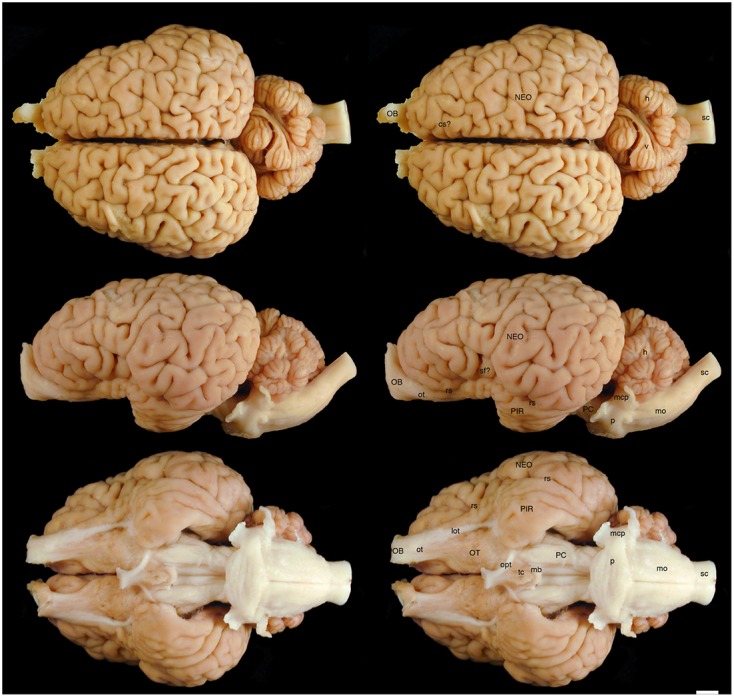
Dorsal, lateral, and ventral views of the brain of the black rhinoceros (*Diceros bicornis*). Left column of images unlabelled. Right column of images, duplicates of those on the left with a number of specific structures labeled. Note the typically mammalian appearance of the brain in terms of the major anatomical subdivisions. Also note the very asymmetrical appearance of the vermal portion of the cerebellum. Scale bar = 1 cm. **cs?,** potential cruciate sulcus; **h,** hemispheric portion of cerebellum; **lot,** lateral olfactory tract; **mb,** mammillary bodies; **mcp,** middle cerebellar peduncle; **mo,** medulla oblongata; **NEO,** cerebral neocortex; **OB,** olfactory bulb; **opt,** optic tract; **OT,** olfactory tubercle; **ot,** olfactory tract; **p,** pons; **PC,** cerebral peduncle; **PIR,** piriform cortex; **rs,** rhinal sulcus; **sc,** spinal cord; **sf?,** potential sylvian fissure; **tc,** tuber cinereum; **v,** vermal portion of cerebellum.

**FIGURE 2:**
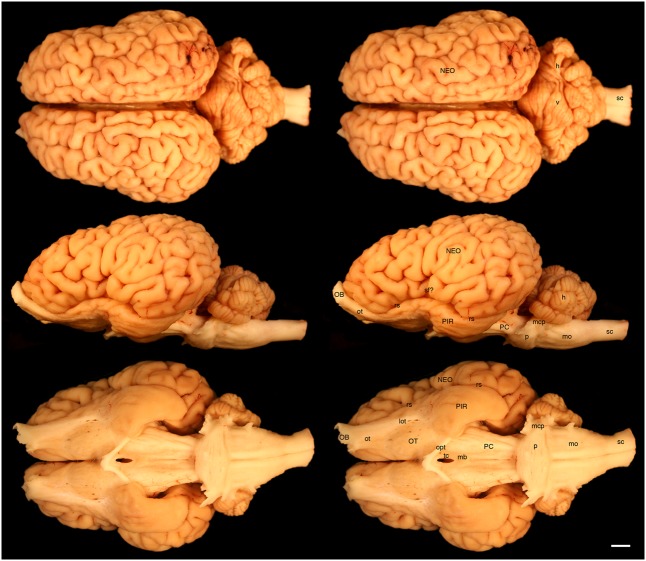
Dorsal, lateral, and ventral views of the brain of the white rhinoceros (*Ceratotherium simum*). Left column of images unlabelled. Right column of images, duplicates of those on the left with a number of specific structures labeled. Note the typically mammalian appearance of the brain in terms of the major anatomical subdivisions. Also note the very asymmetrical appearance of the vermal portion of the cerebellum. Scale bar = 1 cm. **h,** hemispheric portion of cerebellum; **lot,** lateral olfactory tract; **mb,** mammillary bodies; **mcp,** middle cerebellar peduncle; **mo,** medulla oblongata; **NEO,** cerebral neocortex; **OB,** olfactory bulb; **opt,** optic tract; **OT,** olfactory tubercle; **ot,** olfactory tract; **p,** pons; **PC,** cerebral peduncle; **PIR,** piriform cortex; **rs,** rhinal sulcus; **sc,** spinal cord; **sf?,** potential sylvian fissure; **tc,** tuber cinereum; **v,** vermal portion of cerebellum.

The various subdivisions of the rhinoceros brain are clearly demarcated, and follow the organizational pattern generally observed in other mammals (**Figures 1**, **2**). Briefly, two moderately sized olfactory bulbs are found anterior to the two distinct and gyrencephalic cerebral hemispheres. Caudal to the hemispheres the cerebellum, with a distinctly asymmetrical vermal portion, overlies the brainstem (midbrain, pons, and medulla oblongata). In general, the superficial appearance of the black and white rhinoceros brains is very similar, but the black rhinoceros brain appears to be shorter rostrocaudally, wider mediolaterally, and taller dorsoventrally than the white rhinoceros brain (**Figures 1**, **2**). This gives the black rhinoceros brain a mild globular appearance in comparison to the while rhinoceros brain, which appears more elongated in the rostrocaudal dimension. We measured the cerebral hemisphere of each rhinoceros specimen to compared this impression and found that the cerebral hemisphere in the black rhinoceros was 9.3 cm long in the rostrocaudal dimension, compared to 10.7 cm in the white rhinoceros. Dorsoventrally, the black rhinoceros cerebral hemisphere was 7.1 cm, while that of the white rhinoceros was 6.7 cm, and mediolaterally, the black rhinoceros hemisphere measured 10.4 cm, while that of the white rhinoceros measured 10 cm. Thus, these measurements confirm the qualitative impression. However, as we only have one specimen from each species, this may be an individual difference rather that a species-specific difference, but the variation between these two individuals is quite clear. Despite this slight variation in global appearance, both species show similar overall morphology, thus, the description provided herein applies to both species, except where noted.

The most rostral neural structures were the paired olfactory bulbs, which arch dorsally anterior to the rostral pole of the cerebral hemisphere (**Figures 1**, **2**). Unfortunately, the olfactory bulbs of the rhinoceroses are very difficult to remove together with the brain due to the presence of a thick and tough fold of the dura mater between the caudal aspect of the olfactory bulb and the rostral pole of the cerebral hemisphere. Indeed, the drawings of the brain of the Javan rhinoceros provided by Beddard and Treves (1887, plate 37) does not show any olfactory bulbs or even remnants of the bulbs and olfactory tract, with this region of the brain “smoothed” over in the drawings. The olfactory bulbs do not appear to be either overly large, or reduced in size. From the ventrocaudal aspect of the olfactory bulbs a wide (around 15 mm mediolateral) olfactory tract courses caudally toward the brain. This tract coalesces anterior and lateral to the olfactory tubercle (which evinces the distinct arterial spaces that give the name perforated substance) to form a distinct lateral olfactory tract. The lateral olfactory tract appears to invest into a clearly demarcated piriform lobe, which is separated from the neocortex by a distinct rhinal sulcus (which can be followed from the olfactory bulb caudally). The piriform cortex is observed lateral to the olfactory tract, expanding caudally, to form a distinct bulge on the ventral aspect of the cerebral hemisphere. Small sulci and gyri are observed throughout the piriform cortex. Although not quantified, it appears that the piriform lobe of the black rhinoceros is somewhat larger than that of the white rhinoceros, and is also seemingly more gyrencephalic (**Figures 1**, **2**).

**FIGURE 3:**
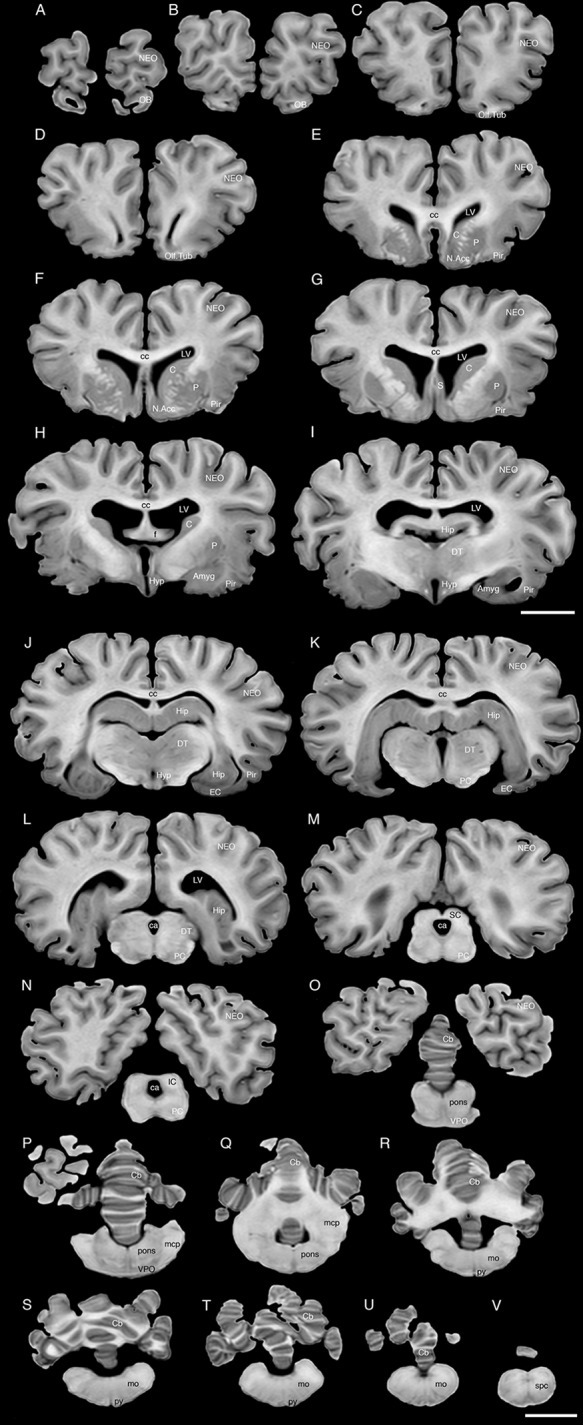
A series of coronal structural MR images through the brain of the black rhinoceros (*D. bicornis*). **(A)** is the most rostral section and **(V)** is the most caudal section, with each section having a thickness of 2 mm and each section being 6 mm apart. Note the typically mammalian topography of the various regions and structures of the brain. Scale bar = 2 cm. Amyg, amygdaloid body; C, caudate nucleus; ca, cerebral aqueduct; Cb, cerebellum; cc, corpus callosum; DT, dorsal thalamus; EC, entorhinal cortex; f, fornix; Hip, hippocampus; Hyp, hypothalamus; IC, inferior colliculus; LV, lateral ventricle; N.Acc, nucleus accumbens; NEO, neocortex; mcp, middle cerebellar peduncle; mo, medulla oblongata; OB, olfactory bulb; Olf. Tub, olfactory tubercle; P, putamen; PC, cerebral peduncle; Pir, piriform cortex; py, pyramidal tract; S, septal nuclear complex; SC, superior colliculus; spc, cervical spinal cord; VPO, ventral pontine nucleus.

**FIGURE 4:**
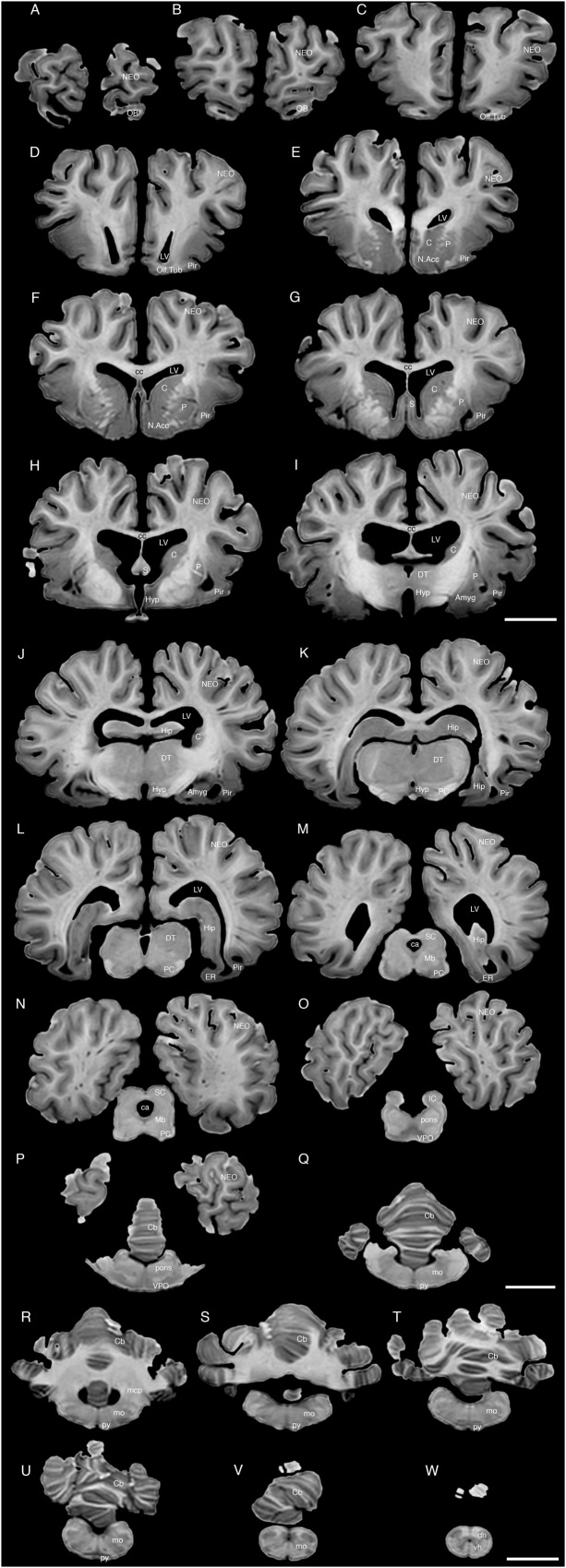
A series of coronal structural MR images through the brain of the white rhinoceros (*C. simum*). **(A)** is the most rostral section and **(W)** is the most caudal section, with each section having a thickness of 2 mm and each section being 6 mm apart. Note the typically mammalian topography of the various regions and structures of the brain. Scale bar = 2 cm. Amyg, amygdaloid body; C, caudate nucleus; ca, cerebral aqueduct; Cb, cerebellum; cc, corpus callosum; dh, dorsal horn; DT, dorsal thalamus; EC, entorhinal cortex; Hip, hippocampus; Hyp, hypothalamus; IC, inferior colliculus; LV, lateral ventricle; N.Acc, nucleus accumbens; NEO, neocortex; Mb, midbrain; mcp, middle cerebellar peduncle; mo, medulla oblongata; OB, olfactory bulb; Olf. Tub, olfactory tubercle; P, putamen; PC, cerebral peduncle; Pir, piriform cortex; py, pyramidal tract; S, septal nuclear complex; SC, superior colliculus; vh, ventral horn; VPO, ventral pontine nucleus.

**FIGURE 5:**
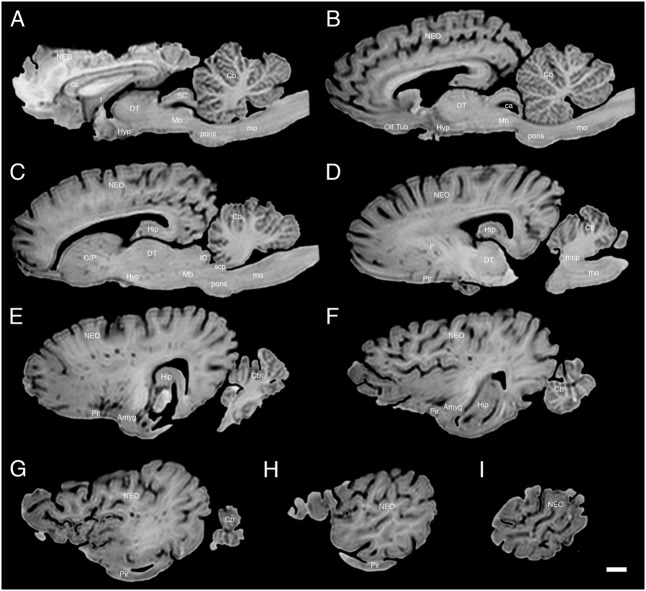
A series of sagittal structural MR images through the brain of the white rhinoceros (*C. simum*). **(A)** is the most medial section and **(I)** is the most lateral section, with each section having a thickness of 2 mm and each section being 6 mm apart. Note the typically mammalian topography of the various regions and structures of the brain. Scale bar = 2 cm. Amyg, amygdaloid body; C, caudate nucleus; ca, cerebral aqueduct; Cb, cerebellum; cc, corpus callosum; DT, dorsal thalamus; f, fornix; Hip, hippocampus; Hyp, hypothalamus; IC, inferior colliculus; NEO, neocortex; Mb, midbrain; mcp, middle cerebellar peduncle; mo, medulla oblongata; Olf. Tub, olfactory tubercle; P, putamen; Pir, piriform cortex; SC, superior colliculus; scp, superior cerebellar peduncle.

**FIGURE 6:**
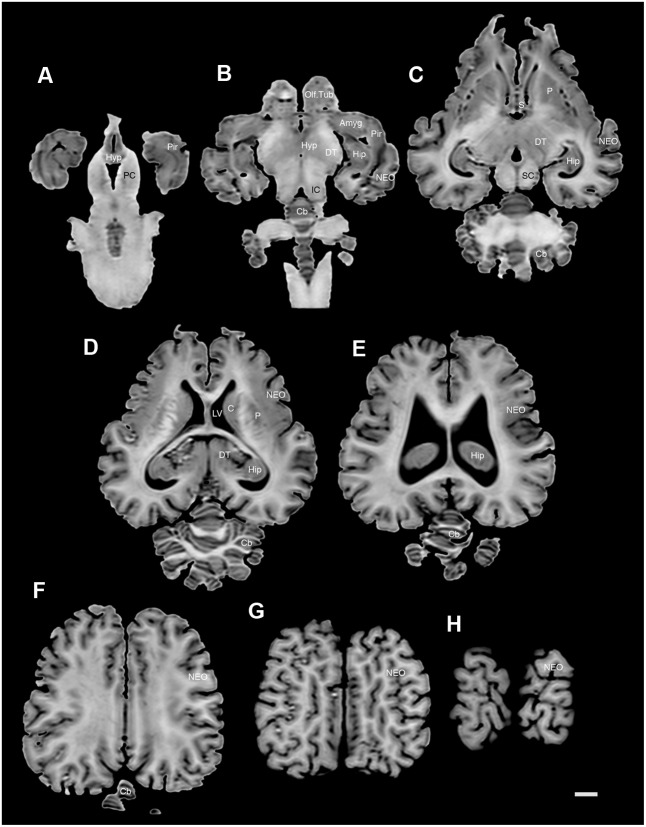
A series of horizontal structural MR images through the brain of the black rhinoceros (*D. bicornis*). **(A)** is the most ventral section and **(H)** is the most dorsal section, with each section having a thickness of 2 mm and each section being 6 mm apart. Note the typically mammalian topography of the various regions and structures of the brain. Scale bar = 1 cm. Amyg, amygdaloid body; C, caudate nucleus; Cb, cerebellum; DT, dorsal thalamus; Hip, hippocampus; Hyp, hypothalamus; IC, inferior colliculus; LV, lateral ventricle; NEO, neocortex; Olf. Tub, olfactory tubercle; P, putamen; PC, cerebral peduncle; Pir, piriform cortex; SC, superior colliculus.

The neocortex occupies the majority of the lateral, dorsal, and mesial surfaces of the brain. The neocortex has numerous sulci and gyri, and these are generally not symmetrical in appearance, i.e., the left and right hemispheres show very different patterns. The majority of the gyri have a width of around 5–7 mm and the sulci do not appear to form long continuous furrows in any plane. There does not appear to be any specifically distinct sulci or gyri that can be conclusively compared to those present in other species, even closely related species such as horses. In addition, our MR images did not assist in the determination of specific sulci and gyri, as many of the sulci are quite deep and do not appear to have enough continuity to allow a specific nomenclature to be applied to them for comparison (**Figures 3**–**6**). In order to avoid confusion, and the potential incorrect assignation of associated functional regions, we have thus not named the sulci and gyri present in the neocortex of the rhinoceros, which was also avoided in earlier descriptions of the brains of other rhinoceros species (Owen, 1850; Garrod, 1878; Beddard and Treves, 1887). While one may be tempted to label a sylvian fissure in the lateral view of the brain of both species (**Figures 1**, **2**) and a possible cruciate sulcus in the rostral dorsal aspect of the black rhinoceros brain (**Figure 1**), at best these identifications would be very tentative.

In our whole brain specimens only the most ventral aspect of the diencephalon is visible, as is typical for mammals (**Figures 1**, **2**). The rostral border of the diencephalon is demarcated by the presence of the optic chiasm and optic tracts. These are not large in size, commensurate with the low number of retinal ganglion cells in the rhinoceroses (Pettigrew and Manger, 2008; Coimbra and Manger, 2017); however, it should be noted that the left optic tract of the black rhinoceros (**Figure 1**) is smaller than the right optic tract, and this appears to be associated with the loss of an eye from this male black rhinoceros 5 years prior to euthanasia following a fight with another male black rhinoceros. Caudal to the optic tracts in the black rhinoceros a distinct tuber cinereum, with associated ventricular space can be observed (**Figure 1**), while in the white rhinoceros, the tuber cinereum appears to have been lost during removal of the brain and the third ventricle is apparent (**Figure 2**). Caudal to the tuber cinereum in the black rhinoceros is a distinct swelling demarcating the caudal aspect of the diencephalon, which appears to be the mammillary bodies (**Figure 1**). The mammillary bodies, while present, appear to be less distinct in the white rhinoceros (**Figure 2**).

Continuing caudally from the diencephalon on the ventral surface of the brain, the floor of the midbrain is represented by rostrocaudally elongated cerebral peduncles with a distinct and also elongated interpeduncular fossa clearly visible, as well as the ventral median sulcus (**Figures 1**, **2**). In keeping with the comparatively elongated nature of the white rhinoceros brain compared to the black rhinoceros brains, the cerebral peduncles are longer in the rostrocaudal direction in the white rhinoceros. At the caudal end of the cerebral peduncles the ventral aspect of the pons bulges ventrally from the surface and exhibits the typical mediolaterally oriented fiber pathways, or stria, typical of mammals. These stria coalesce laterally to form the distinct middle cerebellar peduncles which enter the ventral aspect of the cerebellum. Emerging from the lateral aspect of the pons is the root of the trigeminal nerve, while the caudal border of the pons is marked by the coalesced roots of the facial and vestibulocochlear nerves (**Figures 1**, **2**). Caudal to the pons, a broad medulla oblongata is observed, with visible pyramidal tracts and small laterally placed bulges representing the inferior olivary nuclear complex. A distinct ventral median sulcus that continues into the spinal cord is also present. The medulla oblongata tapers toward the spinal cord, becoming less broad caudally until the spinomedullary junction is reached, although this junction is not clearly marked by the decussation of the pyramidal tracts. The cephalic end of the cervical spinal cord appears to maintain a consistent cross-sectional area for at least 20 cm caudal to the foramen magnum, which is where we sectioned through the spinal cord to remove the brain.

The dorsal surface of the brainstem is completely covered by the cerebellum (**Figures 1**, **2**). The cerebellum shows clear folia and is slightly broader mediolaterally in the white rhinoceros compared to the black rhinoceros. A distinct flocculus is not apparent, but the vermal and hemispheric portion of the cerebellum can be seen. Interestingly, in both species the vermis, while being located at the midline, is highly asymmetrical and forms asymmetrical borders with the cerebellar hemispheres. Indeed, the cerebellum of both species appears somewhat loosely and asymmetrically organized when compared to many other mammalian species.

### Internal Structures of the Brain Apparent in MR Images

The MR images reveal substantial detail regarding the internal organization of the brains of both species of rhinoceros and, as for the external surface of the brain, the organization of the internal aspects of the brain revealed with MR imaging are similar between the two species (**Figures 3**–**6**). Due to this similarity, the following description applies to both species unless otherwise stated. For the most part, the internal organization of the rhinoceros brain is what would be considered typically mammalian in that the regular subdivisions of the brain are apparent. The remnants of the olfactory bulbs are evident in the MR images, and within these remnants a distinct olfactory ventricle is found. At its caudal most aspect the olfactory ventricle communicates with the lateral ventricle via a small duct. Although the olfactory tract is not readily visible in the MR images, the olfactory tubercle and piriform cortex are apparent. The piriform cortex can be followed caudally where it expands ventrally to cover the ventral aspects of both the amygdaloid and hippocampal complexes.

The MR images reveal the depths of the numerous sulci, many of which can be up to 2 cm deep. The images confirm the difficulty in the precise identification of sulci and gyri by examining the surface of the brain and unfortunately do not provide additional information. Within the telencephalon, the corpus striatum, septal nucleus complex, basal forebrain, amygdaloid body, hippocampus, claustrum, and lateral ventricles were readily observed. The corpus striatum occupied the position typically found in mammals, with a clear head of the caudate nucleus being observed rostral to the splitting of this nucleus into the caudate and putamen by the internal capsule. The body of the caudate nucleus could be followed caudally along the dorsal lateral wall of the lateral ventricle (**Figures 3E–H**, **4E–J**, **5C,D**, **6C,D**). No clear globus pallidus was observed with the MR images. The septal nuclear complex is apparent as a small gray matter bulge on the medial wall of the cerebral hemisphere at the level of the decussation of the anterior commissure (**Figures 3G**, **4G,H**). No clear subdivisions of the septal nuclear complex were evident in the MR images. Ventral to the septal nuclear complex, between it and the olfactory tubercle the region where the diagonal band of Broca should be found histologically was observed in the MR images, and immediately lateral to this was the nucleus accumbens (located just anterior and ventral to the head of the caudate nucleus). Immediately caudal and ventral to the posterior pole of the putamen, within the medial aspect of the temporal lobe, the amygdaloid complex could be observed. Both nuclear and cortical portions of the amygdaloid nucleus could be seen, but greater detail in terms of the subdivisions of the amygdaloid complex were not visible (**Figures 3H,I**, **4I,J**, **5E,F**, **6B**). The hippocampus occupied a typically mammalian position within the telencephalon (**Figures 3J–L**, **4K–M**, **5C–F**, **6B–E**), with the temporal pole of the hippocampus located caudal to the amygdaloid complex. The amygdaloid complex and the temporal horn of the hippocampus are separated by an incursion of the lateral ventricle. A distinct vertically oriented ventral portion of the hippocampus located lateral to the diencephalon, before turning forward to form the dorsal portion of the hippocampus lying over the diencephalon. From the most rostral part of the dorsal hippocampus the fornix was readily seen to turn ventral and invest into the hypothalamus and mammillary bodies. The lateral ventricles occupied a position typical of mammals within the telencephalon, having a frontal horn (connected to the olfactory ventricle), a body overlying the corpus striatum and diencephalon, a small temporal horn dorsal to the hippocampal formation in the temporal lobe, but no clear occipital horn could be observed. The ventricles of the white rhinoceros appear somewhat larger than those of the black rhinoceros (**Figures 3**–**6**).

The diencephalon is found in the caudal ventral half of the cerebral hemisphere, and is united across the midline by a large interthalamic adhesion. The rostral border of the diencephalon coincides with the optic chiasm, while the caudal border coincides with the appearance of the superior colliculi of the midbrain (**Figures 3H–M**, **4H–M**, **5A–D**, **6A–C**). The dorsal thalamus, hypothalamus and parts of the epithalamus and ventral thalamus are evident in the MR images. The dorsal thalamus appears as a continuous large cylinder of gray matter extending the entire rostrocaudal length of the diencephalon. No clearly demarcated internal medullary lamina was evident in the MR images, prohibiting further parcellation of the dorsal thalamus into component parts. The hypothalamus is found below the dorsal thalamus, and for the most part surrounding the lower portions of the third ventricle. The caudal border of the hypothalamus is marked by the presence of the mammillary bodies, and does not extend the full rostrocaudal extent of the diencephalon. The fornix can be observed in the hypothalamus, dividing the hypothalamus into approximately equal sized medial and lateral zones (**Figure 3I**). The only portions of the epithalamus apparent in the MR images are the two habenular complexes located dorsal and caudal to the dorsal thalamic nuclear mass.

The MR images reveal the three cerebellar peduncles (middle most clearly), as well as the extent of the foliation of the cerebellar cortex and the size of the cerebellar white matter, but the deep cerebellar nuclei are not apparent (**Figures 3P,Q**, **4P**, **5D**). The asymmetry of the vermal region of the cerebellum is clear in the MR images, but the size and/or extent of the flocculus is inconclusive. Several parts of the midbrain, including the superior colliculi, the inferior colliculi, the cerebral aqueduct, periaqueductal gray matter, midbrain tegmentum, and the cerebral peduncles were readily observed in the MR images (**Figures 3M,N**, **4M–O**, **5B,C**, **6A–C**). Vague gray regions dorsal and medial to the cerebral peduncles may represent the ventral tegmental area and the substantia nigra, but this needs immunohistochemical staining to verify. Within the pons the pontine tegmentum and ventral pontine nuclei can be identified. Rostrally in the medulla oblongata, at the ventral midline, the pyramidal tracts are clearly seen, however, they become less obvious caudally in the medulla oblongata. Occasional patches of gray matter are observed in the medulla oblongata, but to ascribe these to specific structures is difficult without histological verification. Lastly, as the level of the spinomedullary junction, the decussation of the pyramidal tract is apparent (**Figures 3U,V**, **4V**).

### Volumetric Analyses

**FIGURE 7:**
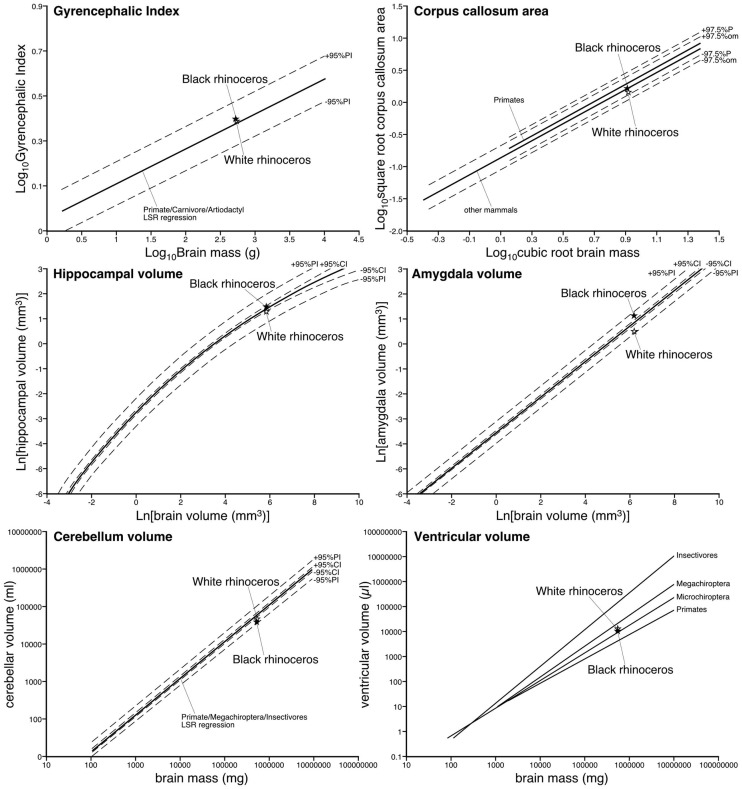
Graphs depicting the relative size of various parameters of the central nervous system in the black and white rhinoceroses in comparison to regressions derived on data from other mammals previously reported. Note that for all parameters measured the relative size of these parameters are close to what would be predicted based on the data from other mammals. Thus, the relative proportions of the brain of the rhinoceroses appear to be typically mammalian.

Using the methodology described in Zilles et al. (1989); Pillay and Manger (2007), and Manger et al. (2012), we calculated the GI of both rhinoceros. For the black rhinoceros we calculated a GI of 2.47, while for the white rhinoceros we found a GI of 2.44. We compared these values to a regression calculated in Manger et al. (2012) based on the GI of primates, carnivores and artiodactyls, and found that both species of rhinoceros had GIs that fell slightly above this regression, but well within the 95% prediction intervals (**Figure 7**; Manger et al., 2012). Thus, the extent of gyrencephaly in the rhinoceros brain appears to be very close to what one would predict for a mammal based on their brain mass. The mid-sagittal cross-sectional area of the corpus callosum of the male black rhinoceros was found to be 2.52 cm^2^, while that of female white rhinoceros was 2.19 cm^2^. These values are very close to what would be predicated based on regressions derived from other non-primate mammals and fall well within the 95% prediction intervals based on the data for other mammals (**Figure 7**; Manger et al., 2010).

The volume of the hippocampus in the black rhinoceros was found to be 4.71 cm^3^, while that of the white rhinoceros was 3.97 cm^3^. The hippocampal volume in both rhinoceros species is very close to what would be predicted based on regressions derived from other mammals, with the black rhinoceros falling within the 95% confidence intervals of the mammalian regression, and both species falling within the 95% prediction intervals of the mammalian regression (**Figure 7**; Patzke et al., 2015). The volume of the amygdala in the black rhinoceros was found to be 3.10 cm^3^, which was markedly larger than that for the white rhinoceros, which had an amygdalar volume of 1.67 cm^3^. Despite this difference in size, the amygdalar volume of both species fell within the 95% prediction intervals based on the regression developed for other mammals, but both fell outside the 95% confidence intervals for this regression (**Figure 7**; Patzke et al., 2015). Interestingly, the black rhinoceros amygdalar volume is slightly larger than you would expect for a mammal with its brain mass, while the white rhinoceros has an amygdalar volume that is somewhat smaller than you would expect for its brain volume. This may be related to the age of the individual specimens, with the black rhinoceros being markedly older than the white rhinoceros, or it may reflect a true species difference.

The black rhinoceros was found to have a cerebellar volume of 37.73 cm^3^, and the white rhinoceros had a cerebellar volume of 44.50 cm^3^. Using a regression based on primate, megachiropteran, and insectivore cerebellar volumes (Maseko et al., 2012), it was found that the volume of the cerebellum for both species of rhinoceros fell within the 95% prediction intervals, but below the 95% confidence intervals (**Figure 7**; Maseko et al., 2012). Thus, in comparison to the species used to create the regression, the size of the rhinoceros cerebellum is on the low side, but within than the range that would be predicted for their brain mass. The total ventricular volume of the black rhinoceros brain was calculated to be 9.86 ml, while in the white rhinoceros the total ventricular volume was found to be 12.05 ml. When comparing these volumes to those obtained in other mammals (Maseko et al., 2011), it is clear that both rhinoceros have total ventricular volumes that fall within the ranges seen for other mammals, having neither a particularly large, or particularly small ventricular system for their respective brain masses (**Figure 7**; Maseko et al., 2011).

## Discussion

The current description and analysis of the gross morphological features of the external and internal aspects of the brains of two species of rhinoceros indicate that the rhinoceros brain is typically mammalian in its general structure, organization, and relative topology of the component parts of the brain. In addition, volumetric analyses of specific aspects of the rhinoceros brains supports this overall conclusion, with no specific regions of the brain investigated being larger or smaller than predicted for a mammal of the brain masses exhibited by the rhinoceroses studied. There were some notable qualitative differences between the two species, with the black rhinoceros having a more globular appearance of the brain, larger mammillary bodies and a larger, more distinct, piriform lobe than the white rhinoceros, while the white rhinoceros had a larger ventricular system than the black rhinoceros. It should also be noted here that the black and white rhinoceros form the African branch of the Rhinocerotidae and despite exact dates not being available for the time of divergence between these two species (although more recent than 20 million years ago, Norman and Ashley, 2000), they are each other’s closest extant relatives (Price and Bininda-Emonds, 2009). In this sense it is not surprising that the general appearance of the brains of these two species is very similar.

### The Typically Mammalian Organization of the Rhinoceros Brain

As outlined, the general appearance of the brain of the two rhinoceros individuals studied is very typically mammalian, which is not a surprising finding. In addition, no specific specializations of the brain were observable at the level of analysis undertaken, and major organizational changes, in regards to proportions of different brain regions, the topological organization and appearance, was limited to the lack of an identifiable cortical sulcal and gyral pattern and the asymmetrical vermal region of the cerebellum in the rhinoceroses. Due to the variance of the sulcal and gyral pattern across the four hemispheres available for study, variation supported by the MR images, we avoided labeling specific sulci and gyri as this might infer functional aspects of the cerebral cortex that may be incorrect. Indeed, Garrod (1878, p. 411) states: “So complicated and numerous are the convolutions that the general type-plan of their disposition is to a considerable extent disguised.” This lack of a clear pattern in the anatomy of the sulci and gyri appears to be specific to the rhinoceroses, as several sulci and gyri that are apparently homologous to those in other mammalian species, are readily apparent on the horse brain (Edinger, 1948; Pascalau et al., 2015). In addition, the vermal region of the rhinoceros cerebellum is very asymmetrical, which is also not often seen across mammalian species, and horses show a distinctly symmetrical vermis (Bradley, 1899). This would mean that a full mid-sagittal section through the vermis of the rhinoceroses would not produce the classical arbor vitae appearance of the cerebellum. This vermal asymmetry is more marked in the black rhinoceros specimen than the white rhinoceros specimen. Interestingly, in their depictions of the Sondiac rhinoceros brain (Beddard and Treves, 1887, plate 37) and Sumatran rhinoceros brain (Garrod, 1878, plate LXX) the authors show a very symmetrical cerebellum, indicating that potentially the asymmetrical vermis is a derived feature of the African rhinoceroses, although as noted for the olfactory bulbs, these diagrams appeared to be somewhat enhanced compared to the anatomical reality of the rhinoceros brain. Despite these two variations, the remainder of the rhinoceros brains can be readily navigated by those with a basic understanding of the gross anatomy of larger mammalian brains.

### Qualitative Differences between Black and White Rhinoceros Brains

In the current analysis we noted four specific qualitative differences between the brain of the black and white rhinoceros investigated, including the more globular appearance of the black rhinoceros brain, the larger mammillary bodies and piriform lobe of the black rhinoceros brain, and the larger ventricular system of the white rhinoceros brain. While we note these differences as differences between the species generally, the strength of the emphasis we place on these differences must be measured by the fact that we only have one brain of each species, therefore the differences might be those of individuals rather than species. However, given that the acquisition of these brains was truly random (in that there were no selection criteria for the specific individuals apart from veterinary necessity), it is likely that the individual brains examined are representative of the species as a whole, and therefore the differences are likely to be specific species differences.

It is clear that the brain of the black rhinoceros is shorter rostrocaudally, but broader mediolaterally and taller dorsoventrally than the white rhinoceros brain (**Figures 1**, **2**). This makes the black rhinoceros brain appear somewhat globular in comparison to the white rhinoceros brain. Whether this difference is due to changes occurring in the evolutionary history of the black or the white rhinoceros is unclear – we don’t have a description of the brain of the last common ancestor of these two species to compare to the extant species. Despite this, the skull being shorter and broader in the black rhinoceros, and longer and narrower in the white rhinoceros are considered key indicators of the two genera (Ansell, 1974). Thus, the shape of the brains reflects the overall shape of the skulls, which in turn are thought to reflect the browsing (black rhinoceros) versus grazing (white rhinoceros) feeding habits of the two species. Palaeoneurological examination of fossil specimens of the two lineages may be able to determine whether the brain became shortened, or lengthened, or both during the evolution of the extant African rhinoceros species.

The mammillary bodies of the black rhinoceros formed distinct ventral bulges at the caudal end of the diencephalon, whereas, in the whole brain specimens the mammillary bodies were not easy to discern in the white rhinoceros. The larger size of the mammillary bodies in the black rhinoceros is commensurate with the slightly larger size of the hippocampus in the black rhinoceros (4.71 cm^3^) compared to the white rhinoceros (3.97 cm^3^), despite the black rhinoceros having a slightly smaller brain than the white rhinoceros. The mammillary bodies, which receive strong input from the hippocampus (via the fornix), are known to be heavily involved in the process of spatial memory and the relationship of head position to cognitive spatial maps (Dillingham et al., 2015). This indicates that, for at least the parts we have identified to date, the overall neural navigation system of the black rhinoceros is larger than that of the white rhinoceros. Male white rhinoceroses are territorial with home ranges of 0.75 to 13.9 km^2^, while female white rhinoceroses have larger home ranges of 3 to 45.23 km^2^ (Skinner and Chimimba, 2005). In contrast, male black rhinoceroses are not strictly territorial and as a species have reported home ranges from as small as 0.5 km^2^ to as large as 500 km^2^ (Skinner and Chimimba, 2005). It appears then that the difference in home range size might account for the larger mammillary bodies in the black rhinoceros. In addition, the black rhinoceroses browse on a greater number of plant species, in a more enclosed environment, that vary across the seasons more dramatically than the grasses grazed by the white rhinoceros (Skinner and Chimimba, 2005). Therefore, in their larger home ranges in a denser wooded habitat, it would be more important for the black rhinoceroses to have a better cognitive map of the location and timing of available food sources than is needed for the grazing diet of the white rhinoceroses, and this may underlie the enlargement of the mammillary bodies in the black rhinoceros.

The piriform cortex of the black rhinoceros appears to be around 125% larger and more gyrencephalic than that of the white rhinoceros, indicating that the processing of olfactory information is of more relevance to the black rhinoceros than the white rhinoceros. Unfortunately, as mentioned earlier, the olfactory bulbs were difficult to remove intact along with the brain, meaning that we don’t have a direct comparison of olfactory bulb size to make between the two species to support the difference observed in piriform cortex size. This potentially increased reliance on olfaction is likely linked to the diet, both in number of plants eaten and their seasonal variability (Skinner and Chimimba, 2005), of the black rhinoceros compared to the white rhinoceros.

The last potential interspecies difference noted was the larger size of the ventricular system in the white rhinoceros compared to the black rhinoceros. The ventricular system of the white rhinoceros is 2.19 cm^3^ (or 122%) larger than that of the black rhinoceros, and this is most evident in the lateral ventricles. Again, this difference may be related to the specific diets of the species, with some of the grasses eaten by the white rhinoceros potentially containing enough toxins that would necessitate the need for larger ventricles to assist in the flushing of toxins from the central nervous system. While the ventricular systems of both species of rhinoceros are not larger than that seen in many other mammals, this interspecies difference is readily apparent.

### Future Studies of Rhinoceros Brains

While the brains of these two rhinoceroses are clearly mammalian, and certain differences are apparent between the two individuals at the gross level investigated herein, it is likely that examination at the microscopic level will reveal additional species-specific features, derived features specific to the Rhinocerotidae, and derived features specific to the Ceratomorpha, and/or Perissodactyla, and/or Laurasiatheria. For example, the presence of a parvocellular cluster of orexinergic neurons in the medial hypothalamus in Cetartiodactyls (Dell et al., 2012, 2017a,b) and African elephants (Maseko et al., 2013) is also likely to be present due to the dietary requirements and phylogenetic affinities of the rhinoceroses. Despite this, it is most likely that a large suite of features common to all mammals will be observed, although variations in the proportional and absolute sizes, as well as neuronal numbers are likely to be found. Thus, our intention is to section these two brains coronally and undertake basic neuroanatomical staining (Nissl and myelin) as well as a suite of immunohistochemical stains to reveal specific and non-specific neural systems and neural structures. It is hoped that by doing this we will be able to relate the neuroanatomy of the rhinoceroses to observed behaviors (as briefly touched upon in the previous section), and perhaps make suggestions for future behavioral observations based on our findings. The overall aim of this process is to help develop a better understanding of the rhinoceros brain and behavior to provide information of relevance to both conservation and management of these iconic African species.

## Author Contributions

AB and PM collected the brains. MH undertook the MR imaging of the brains. AB and PM prepared the draft of the manuscript, which was edited by MH.

## Conflict of Interest Statement

The authors declare that the research was conducted in the absence of any commercial or financial relationships that could be construed as a potential conflict of interest.

## Footnotes

1 www.osirix-viewer.com
